# 

**Published:** 1851-04

**Authors:** 


### TO READERS AND CORRESPONDENTS.

Communications intended for publication, and all letters relating to the business of the Journal, or containing remittances in money, should be directed to Drs. Harris and Blandy, Baltimore, Md.

The communication from E. T., showing the inexpediency and impracticability of teaching dentistry by adjunct professors in medical colleges, was received too late for publication in the present number; it shall appear in our next.

The following Publications and Journals have been received:

Report of the Pennsylvania Hospital for the Insane, for the year 1850. By Thomas S. Kirkbridge, M. D. Philadelphia, 1851.

Ether and Chloroform: their Employment in Surgery, Dentistry, Midwifery, Therapeutics, &c. By J. F. B. Flagg, M. D., Surgeon Dentist, Member of the Rhode Island Medical Society. Philadelphia: Lindsay & Blakiston, 1851.

The Medical Student's Guide in Extracting Teeth; with Numerous Cases in the Surgical Branch of Dentistry. With Illustrations. By S. S. Hornor, Practical Dentist. Philadelphia: Lindsay & Blakiston, 1851.

Reports of the President and Resident Physician of the Maryland Hospital, (for the Insane,) for the year 1850. Baltimore: Printed by John D. Toy, corner of Market and St. Paul streets.

Success in the Medical Profession. An Introductory Lecture, delivered at the Massachusetts Medical College, Nov. 6th, 1850. By John Ware, M. D., Hersey Professor of the Theory and Practice of Physic in Harvard University. Boston: David Clapp, Printer, 184 Washington street, Medical and Surgical Journal Office, 1851.

Address before the Graduates of Washington University of Baltimore, at the Twenty-sixth Annual Commencement, held March 3, 1851. By Thos. E. Bond, A. M., M. D. Baltimore: Printed by Sherwood & Co., N. W. corner Baltimore and Gay streets, 1851.

On the Physiological Effects of Sulphuric Ether, and its Superiority to Chloroform. By William T. G. Morton, M. D. Boston: Printed by David Clapp, 184 Washington-st., Med. and Surg. Journal Office, 1850.

The American Journal of the Medical Sciences. Edited by Isaac Hays, M. D., January and April, 1851.

The London Medical Gazette, November, December, 1850, and January, 1851.

The Boston Medical and Surgical Journal. Edited by J. V. C. Smith, M. D., January, February and March, 1851.

The British and Foreign Medico-Chirurgical Review, January, 1851.

The New York Dental Recorder. Edited by C. C. Allen, M. D., Dentist, January, February and March, 1851.

The Dental Register of the West. Edited by James Taylor, M. D., D. D. S., January, 1851.

### *To Readers and Correspondents.*

The Dental News Letter, 1851.

The London Medical Times, November, 1850.

The Buffalo Medical Journal. Edited by AUSTIN FLINT, M. D., January, February and March, 1851.

The Provincial Medical and Surgical Journal. Edited by W. H. RANKIN, M. D., and J. H. WALSH, Esq., December, 1850, January and February, 1851.

The Dublin Medical Press, January and February, 1851.

The British American Medical and Physical Journal. Edited by ARCHIBALD HALL, M. D., January, February and March, 1851. The Nov. No. for 1850, has not been received; will the editor do us the favor to send it.

The Medical Examiner. Edited by F. G. Smith, M. D., January, February and March, 1851.

The Charleston Medical Journal and Review. Edited by D. J. CAIN, M. D., and F. PEYREL PORCHER, M. D., December, 1850, January, February and March, 1851.

The New York Journal of Medicine and the Collateral Sciences. Edited by S. S. PURPLE, M. D., January and March, 1851.

The Southern Medical and Surgical Journal. Edited by J. P. GARVIN, M. D., January, February and March, 1851.

The North-Western Medical and Surgical Journal. Edited by J. EVANS, M. D., and E. G. MEEK, M. D., November and December, 1850, and January and February, 1851.

The New York Medical Gazette, and Journal of Health. Edited by D. MEREDETH REESE, M. D., LL. D., January, February and March, 1851.

The Western Journal of Medicine and Surgery. Edited by L. P. YANDELL, M. D., and T. S. BELL, M. D., January, February and March, 1851.

The St. Louis Medical and Surgical Journal. Edited by M. L. LINTON, M. D., J. S. MOORE, M. D., WM. M. MCPHEETERS, M. D., and J. B. JOHNSON, M. D., January and February, 1851.

The Ohio Medical and Surgical Journal. Edited by S. HANBURY SMITH, M. D., December, 1850, and January, 1851.

The Western Lancet and Hospital Reporter. Edited by L. M. LAWSON, M. D., and GEORGE MENDENHALL, M. D., January, February and March, 1851.

The New Hampshire Journal of Medicine. Edited by EDWARD H. PARKER, A. M., M. D., January, February and March, 1851.

The New Jersey Medical Reporter, and Transactions of the New Jersey Medical Society. Edited by JOSEPH PARISH, M. D., January and February, 1851.

The New York Register of Medicine and Pharmacy. Edited by C. D. GRISWOLD, M. D., January, February and March, 1851.

The London Lancet, Journal of British and Foreign Medical, Surgical and Chemical Sciences, Chemistry, Literature and News. Editor, THOS. WAKELEY, M. D., for London: Sub-Editors, J. H. BENNET, M. D., and T. WAKELEY, M. R. C. S. E., January, February and March, 1851.

